# Gender inequality in incivility: Everyone should be polite, but it is fine for some of us to be impolite

**DOI:** 10.3389/fpsyg.2022.966045

**Published:** 2022-09-26

**Authors:** Xing J. Chen-Xia, Verónica Betancor, Alexandra Chas, Armando Rodríguez-Pérez

**Affiliations:** Department of Cognitive, Social and Organizational Psychology, Faculty of Psychology, University of La Laguna, Santa Cruz de Tenerife, Spain

**Keywords:** civility, gender norms, social norms, social process, stereotypes

## Abstract

Civility is formed by social norms that guide our behavior and allow us to interact appropriately with others. These norms affect everyone and are learned through the socialization process. However, in the same process, people also learn gender norms that dictate how men and women should behave, leading to gender stereotypes and differentiated behavioral characteristics. The purpose of this research is to examine the relationship between gender and civility, and how we react to those who behave uncivilly given their gender. The results of Study 1 (*N* = 153) showed that even in a fictional and gender-neutral society, uncivil behaviors were associated with stereotypically masculine characteristics, and those who behaved uncivilly were dehumanized. In Study 2 (*N* = 144), gender differences were observed in incivility. Women were harsher when facing uncivil transgressors than men, especially if the transgressor was another woman. Our findings support the notion that gender norms are applied to civility, leading those supposedly equal social norms to unequal perceptions and evaluations.

## Introduction

In modern society, it is common for us to expect civil interaction with others in our daily lives. Social interaction is based on civility, behaviors characterized by courtesy and good manners (Forni, 2002). These behaviors are regulated by social norms that are necessary for survival and to sustain an urban life characterized by unpredictable and transitory relations between strangers (Lofland, 1998; Páramo, 2013). Unfortunately, not all behaviors follow these rules, there are also uncivil behaviors, that are counter-normative or deviant behaviors that may or may not be illegal (Osgood et al., 1996; Nugier et al., 2009), whose presence highly reduces life quality (Robin et al., 2007). It is necessary to follow social norms in order to live together in harmony, and these norms are learned through the process of socialization (Elias and Hammer, 1939). However, in this process we also learn gender norms that dictate the appropriate behaviors for men and women, pressuring them to engage in behaviors based on traditional masculine or feminine norms (Hemsing and Greaves, 2020).

Precisely, the purpose of our research is to examine the relationship between gender and civility, and how we react to those who transgress the norms of civility. The social norms that dictate civil and uncivil behaviors may be related differently to men and women, since gender stereotypes may affect how they perceive behaviors that describe a good or a bad citizen, and how they react to them.

Social norms are rules that guide people’s behavior in a given society or group and define what is acceptable and how the members should behave (Cislaghi and Heise, 2018). This includes how people should dress up depending on the occasion, the methods of greeting others, how to behave in public spaces, and interactions with others such as giving up your seat on a public bus, or waiting for your turn to speak in an online seminar. However, these social norms not only vary between cultures (Moon and Sánchez-Rodríguez, 2021), they may be influenced by gender norms that are present in the world outside of the individual too. These norms have existed since a boy or girl is born, and learned, consciously or unconsciously, from parents and peers through socialization while growing up (Bem, 1981; Grusec and Hasting, 2007; Mulvey and Killen, 2015). In this sense, the Social Role Theory (Eagly, 1987) states that the gender division of labor that society has, leads to widely shared gender stereotypes. Following this, in western societies, prevailing stereotypes associate agency with men and communion with women, since men are usually assigned to roles with power and status, and women with nurturant roles. The gendered division of labor in a society leads men and women to have differentiated skills based on their roles, resulting in gender stereotypes that perpetuate this cycle through the expectations of the members of said society to conform to their roles and act as what is expected of them. Gender norms can lead to human differentiation based on stereotypes. This differentiation can affect how people process information and categorize stimuli, even leading them to associate entities that have no gender, with men (masculine) or women (feminine) (Martin and Slepian, 2018).

In this research, our first aim is to test whether those who perform uncivil and civil behaviors are associated more with stereotypically masculine or feminine characteristics, as well as how the lack of civility may trigger dehumanization as a consequence of not following social norms. There is an extensive body of literature showing an asymmetrical attribution of stereotypically masculine and feminine characteristics (e.g., White and Gardner, 2009; Ebert et al., 2014). This difference is stated in the Social Role Theory (Eagly, 1987) where men are more related to agency and women to communion. It is also observed in the stereotype content model (SCM) of Fiske et al. (2002), where they state that masculinity is related to competence, whereas femininity is more related to warmth. More importantly, this difference between men and women is not innate but learned. Andersen et al. (2013) compared the competitiveness of children in matrilineal and patriarchal villages in India, and they observed that in patriarchal societies women become less competitive through the process of socialization, but not in matrilineal societies (Andersen et al., 2013). Therefore, in patriarchal societies, such as the Spanish one, which is what interests this work, women learned that competence is not a stereotypically feminine trait, so they became less competent, which is a consequence that can lead to negative outcomes for them such as future job prospects or personal capability. This is relevant since several studies showed that perceiving someone as less competent could lead to attributing less humanity to this person (Jones-Lumby and Haslam, 2005; Vaes and Paladino, 2009; Rodríguez-Pérez et al., 2021).

Civility is a uniquely human trait (Haslam, 2006), the ability to learn these social norms and self-regulate our behaviors to facilitate a healthy social interaction with others is a characteristic that differentiates humans from animals, so a lack of civility may trigger animalistic dehumanization. Behaving uncivilly is related to primitive beings that lack refinement, morality, and a sense of community, so those who behave in this way can be seen as less human (Saminaden et al., 2010). Being dehumanized is a serious concern since it has been prevalent in intergroup violence, justifying the aggressive actions committed to those considered as less human, leading people to support torture and violence toward the dehumanized ones (Kteily et al., 2015). This highlights the importance to study the association between civil and uncivil behaviors that are present in our daily lives, with stereotypically masculine and feminine characteristics, since these associations can lead men and women to behave civilly or uncivilly, and they may trigger devastating consequences such as dehumanization if they behave uncivilly.

The association of a type of behavior with certain traits can lead those who possess said traits to carry these behaviors (Heyder et al., 2021). In this way, following Ellemers (2018), the differences in the association of stereotypically masculine and feminine characteristics can lead to expectations toward men and women about how they should be, reinforcing gender roles, justifying the inequality between both sexes, impacting the way they behave, and influencing how they react to others’ behavior given their gender. Therefore, the second aim of this study is to observe how gender differences may affect the way men and women react to those who carry uncivil behaviors based on the gender of the transgressor.

Research has shown how various social behaviors are affected by gender. For example, Vicente-Molina et al. (2018) examined how a set of daily pro-environmental behaviors were affected by gender. Particularly, they observed that women are more focused on these behaviors than men, stating that pro-environmental behaviors seem to be moderated by gender. These differences are not only present in pro-environmental positive behaviors but also in negative ones, which is of interest to us. There is a body of research on incivilities related to gender which includes court employees (Cortina et al., 2001), attorneys (Cortina et al., 2002), and academia (Richman et al., 1999), and all these studies found that women experience higher rates of uncivil treatment than males did. However, one of the fields of study that has the largest body of research on gender and incivility is the one that focuses on the workplace. In this field, two opposing theories can offer some clues for the present study. Specifically, Cortina (2008), alludes that men constitute the dominant majority and it could give them a sense of threat, and hostility toward women in their outgroup members’ condition. On the other hand, Sheppard and Aquino (2017) support that women commonly experience incivility from other women because they can perceive them as different and have to compete with them. Interestingly, Gabriel et al. (2018) found evidence to support that women who violate the gender stereotypes (more agentic) have more possibilities that other women behave uncivil toward them but they do not find effects for communion.

Along the same line, Carmona-Cobo et al. (2019) studied the perception and acceptance of incivilities in a male-dominated profession like engineering. They found that women were more aware and accepted less incivility than men in general, whereas men showed more awareness and accepted less incivility when it came from a woman than another man. However, these inconsiderate, rude, and humiliating behaviors were researched in specific contexts such as the workplace (Vega and Comer, 2005; Sutton, 2007; Tepper, 2007), and in academic and school centers (Alberts et al., 2010), where the expectations about their behavior could be more influenced by their role in that institution than their gender (Eagly, 1987). Also, it was usually studied with the behaviors that are specific to these contexts, but incivility is also present outside of these contexts and job-related roles, manifested with general uncivil behaviors that were less studied, by people whose role is being a citizen and where the gender stereotypes have a higher impact on their behavior (Eagly, 1987). Based on this, Barnett et al. (2005) conducted a study to verify the factors associated with people’s provision to engage in several miscellaneous minor moral and legal violations and the role of gender differences concerning such behaviors. Their results showed that participants expected more male than female college students to engage in minor moral and legal violations, also, those male participants reported more willingness of engaging in transgressions than female participants, especially in transgressions that involve elevated risk-taking. However, there is scarce literature focused on the relation between incivility and stereotypical gender traits’ perceptions in an everyday context. Thus, it is in our interest to observe how men and women react to people that perform daily incivilities that transgress social norms, based only on the gender of the transgressor. This study pretends to extend the current literature on gender norms and incivility in general contexts in different ways. First, it seeks to determine the relationship between gender, civility, and dehumanization. Second, it tries to contribute to the stereotypes research and their consequences on dehumanization and civility. And third, it tries to offer more evidence to the literature on uncivil behaviors in everyday life.

## Study 1

The purpose of the first study is to explore the associations between those who carry civil and uncivil behaviors, with stereotypically masculine and feminine characteristics, and how the lack of civility may trigger the dehumanization of the uncivil agent.

The difference in socialization between men and women is present across cultures. Generally, women are socialized to be more expressive, interdependent, caring, compassionate to others, cooperative, communal, and socially responsible (Chodorow, 1974; Gilligan, 1982; Eagly, 1987; Beutel and Marini, 1995) traits that are related to civil behaviors. Whereas, men are socialized to be more independent, masterful, rational, agentic, and competitive (Keller, 1985; Eagly, 1987; Creighton and Oliffe, 2010). Moreover, males also report a higher likelihood to carry minor moral and legal violations than females, traits that are related to uncivil behaviors. And, not only do they perform these transgressions more than females, males are also expected to engage in more minor moral and legal violations than females (Barnett et al., 2005). With this in mind, we propose that uncivil behaviors, as a type of transgression that is usually performed and expected from males, will be associated more with stereotypically masculine characteristics than stereotypically feminine ones (Hypothesis 1a). On the other hand, those who carry civil behaviors will be more associated with stereotypically feminine characteristics given their communal nature (Hypothesis 1b). Also, taking into account that civility is a uniquely human trait (Haslam, 2006), we expect that those who perform uncivil behaviors will be dehumanized, that is, be seen as closer to animals than humans given their lack of civility as transgressors of social norms (Hypothesis 1c). Finally, since we hypothesize stereotypically masculine and feminine characteristics to be given asymmetrically to uncivil and civil agents, and for uncivil agents to be dehumanized, we also expect these stereotypically masculine and feminine characteristics to mediate in the dehumanization of the uncivil agent (Hypothesis 1d).

### Materials and methods

#### Participants and design

A total of 153 Spanish undergraduate psychology students participated in this study and gave their informed consent. Participant gender was largely balanced between females (54.9%) and males (45.1%). Their ages ranged from 18 to 53 (*M* = 20.0; SD = 6.7). The students were randomly assigned to each experimental condition (Civil vs. Uncivil vs. Neutral) and were awarded course credit for participating.

The study followed a single factor between-subjects design with the independent variable being the type of behavior with three levels (Civil vs. Uncivil vs. Neutral). Participants were randomly assigned to one of three experimental conditions. In experimental condition one (*n* = 49) participants were presented with an agent performing a civil behavior. In experimental condition two (*n* = 45) they were presented with an agent performing an uncivil behavior. And in experimental condition three (*n* = 59) participants were presented with an agent performing a neutral behavior. Two dependent variables were asked in all conditions: *attributed role* (masculine vs. feminine), and dehumanization *of the agent*. G*Power 3.1 (Faul et al., 2007) suggests we would need 156 participants in order to detect a medium effect size (*f* = 0.25) with 90% power (α = 0.05).

#### Materials

##### Background story

The participants read a text about a member (FFMFMF) from an extraterrestrial society, the Ortandesíes, in which the inhabitants were described as gender-neutral, being neither women nor men, following an approach similar to that adopted by Hoffman and Hurst (1990). The text briefly described this society and affirmed that the members of this society lived in large cities and that they had rules of coexistence and respect for others in order to harmonize life in the community. Furthermore, as in all societies, there were Ortandesíes who behaved civilly and Ortandesíes who behaved uncivilly (see Supplementary Appendix A). After reading the text, participants were presented with FFMFMF performing one behavior (civil, uncivil, or neutral).

##### Type of behavior

We selected two civil behaviors and two uncivil behaviors from Rodríguez-Gómez et al. (Under review)^1^ database of 120 civil and uncivil behaviors evaluated in several dimensions relevant to humanity and civility. Specifically, the two civil behaviors were “Depositing glass in the recycling containers” and “Disconnecting the mobile phone so as not to disturb others.” The two uncivil behaviors were “Not picking up dog’s excrement” and “Damaging street furniture.” In addition, two neutral behaviors not related to civility standards (“Looking at the watch to check the time” and “Talking on the phone”) were included in the design. With these behaviors, a pretest (*N* = 28) was carried out to ensure there would be differences in civility and valence for the civil, uncivil, and neutral behaviors. The questionnaire included a question related to perceived civility (1 = *very uncivil*; 5 = *very civil*) and the valence of behavior (1 = *very negative*; 5 = *very positive*). The repeated measures ANOVA of the three types of behaviors in civility was significant (*F*(2,54) = 108.78; *p* < 0.001; η^2^ = 0.801). Similar results were found for the valence of the behaviors (*F*(2.54) = 247.52; *p* < 0.001; η^2^ = 0.902). The analysis of differences between pairs within the same ANOVA, adjusting for multiple comparisons (Bonferroni) showed that the chosen behaviors for civil, neutral, and uncivil were significantly different in both civility and valence (see Table 1). The two behaviors of each type were randomly distributed among the participants so that each one only read the description of the alien doing one behavior.

**TABLE 1:** Means and standard deviations for each type of behavior in civility and valence.

Type of behavior	Civility		Valence	
	*M*	SD	*M*	SD
Civil	4.21	0.53	4.26	0.39
Neutral	3.00	0.14	3.07	0.22
Uncivil	1.64	0.88	1.54	0.56

##### Masculinity–Femininity scale

Next, participants were asked to indicate on a scale from 1 (*not have this quality at all*) to 7 (*this quality characterizes it very well*) to what extent they consider that a set of stereotypically masculine and feminine characteristics are typical of FFMFMF. Participants were presented with a list of items corresponding to the Spanish adaptation of the Bem Sex Role Inventory (BSRI) [Bem, 1974; adapted into Spanish by Páez and Fernández (2004)]. This inventory consists of 18 items, of which nine measure the social construct of masculinity (α = 0.83)–for example, “strong personality,” “acting as a leader,” and “dominant”–and the other nine items measure the construct of femininity (α = 0.87)–that is, “sensitive to the needs of others,” “loving,” and “loves children” (see Supplementary Appendix A). We expect agents who perform uncivil behaviors to be given more stereotypically masculine characteristics (H1a) and agents who perform civil behaviors to be given more stereotypically feminine characteristics (H1b).

##### Dehumanization of the agent

Participants also responded to a 0–100 horizontal slide question, where 0 = *human* and 100 = *animal*, where they will place the image they formed about the agent, according to the following statement: “When you see FFMFMF perform this behavior, you probably have ideas about FFMFMF. If you had to summarize them at one point on a Human-Animal scale, taking into account the standards of planet Earth, where would you place the image that you have formed?” We expect agents who perform uncivil behaviors to be seen as less human than those who perform civil behaviors (H1c).

#### Procedure and data analysis

We collected data using a self-administered online questionnaire through the Qualtrics platform. To do this, we generated an electronic reference for the survey and distributed it to students through the virtual campus of the university. Participants were asked to carefully read the background story that was presented. After the story, each participant randomly saw a statement where FFMFMF carried one of the six behaviors. The two dependent variables were then presented below.

We used SPSS program 25 version for the analyses. A significance level of.05 was set. Descriptive statistics were calculated, and a mixed-design ANOVA of 3 (Type of behavior: Civil vs. Uncivil vs. Neutral) × 2 (Attributed role: Masculine vs. Feminine), with repeated measures in the last variable, was carried out. Also, a between-subjects ANOVA (one-way) of type of behavior (Civil vs. Uncivil vs. Neutral) as the independent variable was conducted with the dehumanization of the agent as the dependent variable. Also, the SPSS PROCESS macro (Model 4) developed by Hayes (2018) was used to conduct a mediation analysis. All effects were reported with 95% confidence intervals (CIs). The bootstrapping method with 10,000 resamples of the data was used to test the robustness of mediating effects, and all effects were reported with 95% CIs.

### Results and discussion

#### Masculinity–Femininity scale

**FIGURE 1:**
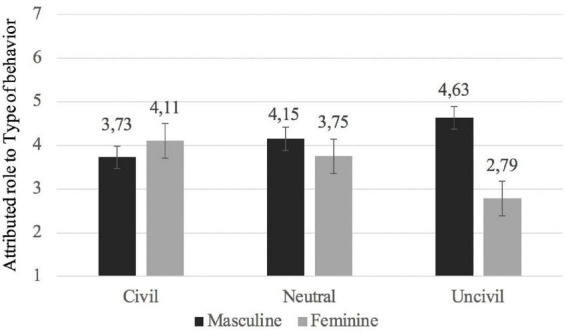
Masculine and feminine characteristics in each behavioral scenario.

To test Hypothesis 1a, we used a mixed-design ANOVA of 3 (Type of the behavior: Civil vs. Uncivil vs. Neutral) × 2 (Attributed role: Masculine vs. Feminine), with Type of behavior as the independent variable and Attributed role as a dependent variable. The results showed a main effect of the role attributed to the alien (*F*(1,150) = 16.33, *p* < 0.001; η*p*^2^ = 0.10). Although participants rated the average alien member as more stereotypically masculine (*M* = 4.17, 95% CI = [3.99, 4.35]) than stereotypically feminine (*M* = 3.55, 95% CI = [3.36, 3.74]), there was a significant interaction between masculinity-femininity and type of behavior, *F*(2,150) = 16.50, *p* < 0.001; η*p*^2^ = 0.18 (see Figure 1).

As seen in Figure 1, there are differences between the stereotypically masculine and feminine characteristics given to others based on their behavior, but this difference did not appear in the same way for the three types of behavior. The analysis of the simple effects of the interaction showed that, in the civil behavior condition, the average extraterrestrial member was evaluated using both stereotypically masculine traits (*M* = 3.73; SD = 1.10) and stereotypically feminine traits (*M* = 4.11; DT = 1.19; *t* (48) = 1.41; *p* = 0.166), which is not in line with Hypothesis 1b. The same occurred in the neutral behavior condition. The alien was described with both stereotypically masculine (*M* = 4.15; SD = 1.23) and stereotypically feminine (*M* = 3.75; *SD* = 1.19; *t* (58) = 1.55; *p* = 0.126) features. However, in the uncivil behavior condition, the average extraterrestrial member was perceived to be more stereotypically masculine (*M* = 4.63; SD = 1.03) than stereotypically feminine (*M* = 2.79; SD = 1.25; *t* (44) = 6.70; *p* < 0.001, *d* = 0.99; 95% CI [0.64, 1.35]). That is, when thinking about uncivil agents, participants associated them with more stereotypically masculine characteristics, which is in line with Hypothesis 1a.

On the other hand, the stereotypical masculine and feminine profiles differed in their association with the types of behavior. The stereotypically feminine profile is higher in the civil behavior condition than in the uncivil behavior condition, (*t* (92) = 5.24; *p* < 0.001; *d* = 0.54, 95% CI [0.32, 0.76]) and the same occurs among the neutral behavior condition and the uncivil behavior condition (*t* (102) = 4.01; *p* < 0.001; *d* = 0.40; 95% CI [0.19, 0.59]). That is, more stereotypically feminine characteristics were given to agents that perform civil and neutral behaviors than agents who perform uncivil behaviors. Interestingly, in the stereotypically masculine profile, there are also differences between the conditions, but these differences appeared in an inverse direction. Specifically, less stereotypically masculine characteristics were given to those who perform civil behaviors than those who perform uncivil behavior condition (*t* (92) = −4.07; *p* < 0.001; *d* = 0.42, 95% CI [0.21, 0.63]), and the same differences appeared between those who perform neutral behaviors and those who perform uncivil behaviors (*t* (102) = −2.12; *p* = 0.036; *d* = 0.21; 95% CI [0.13, 0.40]).

In summary, when comparing stereotypically masculine and feminine characteristics, significant differences between them were found only in the uncivil behavior condition. The one who behaves uncivilly is given more stereotypically masculine characteristics than feminine ones. However, it is interesting that when analyzing the progression of each profile (masculine and feminine) through the three behavioral conditions (civil, neutral, and uncivil), the progression found is significantly inverse. The stereotypically feminine characteristics were the highest in those who carried civil behaviors and lowest in those who carried uncivil behaviors. On the other hand, the stereotypically masculine characteristics were highest in those who behaved uncivilly than in those who behaved civilly. Given this inverse relationship in the progression of the profiles, it is interesting that only hypothesis 1a was confirmed. Differences were found between the uncivil condition and the neutral condition but not between the neutral condition and the civil condition. That is, most stereotypically masculine characteristics and least stereotypically feminine characteristics were given to those who perform uncivil behaviors, which resulted in significant differences. Whereas most stereotypically feminine characteristics and least stereotypically masculine characteristics were given to those who perform civil behaviors, however, this did not lead to significant differences.

#### Dehumanization of the agent

We carried out a between-subjects ANOVA (one-way) of the type of behavior (Civil vs. Uncivil vs. Neutral) with dehumanization of the agent of the behaviors as the dependent variable. Results showed a significant effect (*F*(2,150) = 3.32; *p* = 0.039; η*p*^2^ = 0.042). The paired contrast showed that this significance was due to the difference between the civil agent (*M* = 39.57; DT = 25.91) and the uncivil agent (*M* = 52.91; DT = 23.78; *t* (92) = 2.59; *p* = 0.011; *d* = 0.27, 95% CI [0.06, 0.48]), whereas the neutral agent (*M* = 46.81; SD = 25.47) showed no differences. That is, civil agents are perceived as closer to humans, but uncivil agents are dehumanized and seen as closer to animals, which is in line with Hypothesis 1c.

**FIGURE 2:**
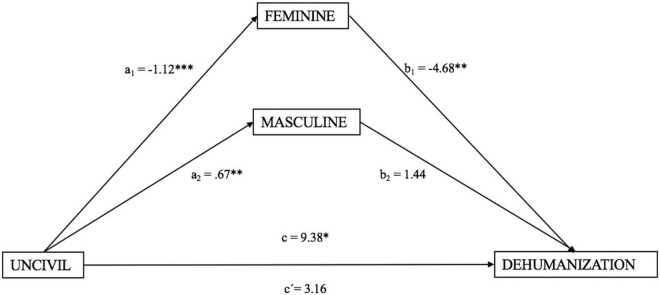
The mediation model of stereotypically feminine characteristics and stereotypically masculine characteristics in the relationship between uncivil agent and dehumanization. Effects were reported as unstandardized values. *p < 0.05; ^**^p < 0.01; ^***^p < 0.001.

In addition, Model 4 in SPSS PROCESS macro was used to test the mediation effect of stereotypically feminine and masculine characteristics on the dehumanization of uncivil agents (see Figure 2).

As seen in Figure 2, uncivil agent was negatively associated with stereotypically feminine characteristics (a1 = −1.12, *p* < 0.001), which, in turn, was negatively associated with dehumanization (b1 = −4.68, *p* = 0.008); whereas, uncivil agent was positively associated with stereotypically masculine characteristics (a2 = 0.67, *p* = 0.001), which, in turn, was negatively associated with dehumanization (b2 = 1.44, *p* = 0.434); meanwhile, the direct effect of uncivil agents on dehumanization was not significant (c′ = 3.16, *p* = 0.510); whereas the total effect of uncivil agents on dehumanization was significant (*c* = 9.38, *p* = 0.038).

To test the indirect effects, we inspected the bootstrapped CIs with 10,000 samples (see Table 2). The total indirect effect of uncivil agent on dehumanization was significant (*B* = 6.23, SE = 2.20, 95% CI [2.27, 10.88]). Specifically, uncivil agents indirectly affected dehumanization through the mediating pathway of stereotypically feminine characteristics (*B* = 5.26, SE = 2.01, 95% CI [1.76, 9.65]), accounting for 56.1% of the total effect.

**TABLE 2:** Stereotypically feminine and masculine characteristics in the mediation analysis.

Effect	*B*	SE	Bootstrapping 95% CI
Total effects	9.38	4.48	[0.54, 18.23]
Direct effect	3.16	4.78	[−6.29, 12.60]
Total indirect effect	6.23	2.20	[2.27, 10.88]
Indirect effect (X → M1 → Y)	5.26	2.01	[1.76, 9.65]
Indirect effect (X → M2 → Y)	0.97	1.24	[−1.59, 3.48]

Based on 10,000 bootstrap samples; Total, direct, and indirect effects of uncivil agents (X) on dehumanization (Y) through stereotypically feminine characteristics (M1) and stereotypically masculine characteristics (M2). SE, standard error; CI, confidence interval.

The results of this study are in line with what we expected. Uncivil behaviors, as social norm transgressions, are associated more with stereotypically masculine characteristics (Hypothesis 1a), and those who behave uncivilly are dehumanized (Hypothesis 1c). However, it is concerning that the differences appeared in the uncivil behavior condition but not in the civil behavior condition, given that femininity was the significant mediator of the dehumanization of uncivil agents. That means those who behave uncivilly will be dehumanized, but this is only predicted through the lack of stereotypically feminine characteristics. In other words, the more uncivilly you behave, the more stereotypically masculine you are, but dehumanization won’t happen because of an excess of stereotypically masculine traits but because of a lack of stereotypically feminine characteristics (Hypothesis 1d). This raises a concern about gendered expectations in our society.

It seems that these everyday social norms that are supposed to be followed by all members of our society, regardless of their gender, are not equally associated with stereotypically male and female characteristics. This difference may be caused by the larger engagement of men with minor transgressions, which may be influencing the normalization of these transgressions for them, which can lead to disturbing differences in the consequences men and women face from doing the same behavior. If incivility is associated more with stereotypically masculine characteristics, given men’s engagement with counternormative behaviors, will they judge incivility less severely than women given the association of these behaviors with masculine traits? Will women face stronger rejection when behaving uncivilly than men given the incongruity of these behaviors with feminine traits? To answer these questions, we conducted our second study.

## Study 2

The purpose of the second study is to observe how gender differences may affect the way we react to those who carry uncivil behaviors. Specifically, how do men and women react to incivilities when an uncivil transgressor is a man vs. when it is a woman?

Diverse investigations observed differences in the behaviors carried and experienced based on gender. In organizational contexts, women engaged in less incivility than men (Alexander-Snow, 2004), and it has been seen that women are especially likely to experience uncivil treatment at work, which can affect their wellbeing (Cortina et al., 2001, 2013; Settles and O’Connor, 2014). Also, in public spaces such as the case of street harassment, men harass women more frequently than in the opposite case (Gardner, 1995; Kearl, 2014). Even in educational institutions, undergraduate women report more experiences of interpersonal sexual objectification than men (Swim et al., 2001). It is clear that gender is particularly important and unequally associated with minor violations (Barnett et al., 2005). In this way, men and women go through different experiences as victims and transgressors of incivilities, so the reactions toward incivilities may vary based on their gender. Research on violations has consistently found that transgressors are more likely to downplay the amount of harm caused relative to victims, being the victims those who report more negative consequences of the violation than transgressors (Baumeister et al., 1990; Baumeister, 1997; Baumeister and Campbell, 1999). Not only do victims perceive the transgressions as more harmful, they also react with more anger and moral outrage, even experiencing dislike for a longer period of time after minor transgressions than major ones (Mikula, 1986; Mikula et al., 1998; Darley and Pittman, 2003; Gilbert et al., 2004; Folger et al., 2005). Also, given the asymmetrical experience and reactions between victims and transgressors, victims report a higher need for justice restoration to reestablish the balance (Adams, 2016). Since women don’t downplay the harmfulness of these transgressions, they will see them as deviant behaviors that need to be addressed, thus reacting with social control, telling the perpetrator that the behavior is wrong (Moisuc et al., 2018), seeking an apology from the transgressor, admitting their wrongdoings, or punishing them to regain their control, status and improve their mood (Wenzel et al., 2008; Gollwitzer and Bushman, 2012). Moreover, it has been observed that victims’ search for retributive justice is highly linked to the moral outrage they feel toward the transgression and the dehumanization of said transgressor (Bastian et al., 2013).

Taking this into account, we expect differences based on the gender of the participant (Hypothesis 2a, 2c, and 2d) and the gender of the transgressor (Hypothesis 2b). First, we expect women to be harsher on uncivil transgressors than men, since they are often victims of deviant behaviors (Hypothesis 2a). Second, following our results from study 1, where uncivil behaviors were given more stereotypically masculine traits, and the Role Congruity Theory (Eagly and Karau, 2002), given the congruity of uncivil behaviors with men and incongruity of uncivil behaviors with women, we expect that when the uncivil transgressor is a woman, she will be evaluated more harshly than when the transgressor is a man (Hypothesis 2b). Third, we expect women, as frequent victims of incivilities, to experience more moral outrage than men when facing uncivil transgressors (Hypothesis 2c). And Fourth, we expect a higher need for justice from women than men (Hypothesis 2d). Specifically, we expect that when facing uncivil transgressors, women will evaluate the transgressor as more uncivil and less human than men, given their higher perception of harmfulness as frequent victims. We also expect women to experience more moral outrage (Kennedy and Kray, 2014; Deng et al., 2016) than men, enacting more social control (Moisuc et al., 2018), and expecting more emotions from the transgressor when called out as a sign of admission of their wrongdoings and willingness to change, to restore the justice (Nugier et al., 2007).

### Materials and methods

#### Participants and design

A total of 144 Spanish undergraduate psychology students participated in this study and gave their informed consent. Participant gender was largely balanced between females (52.08%) and males (47.92%). Their ages ranged from 18 to 24 (*M* = 20.25; SD = 3.73). The students were randomly assigned to each experimental condition and were awarded course credit for participating.

The study followed a 2 (Agent of the behavior: Male vs. Female) × 2 (Gender of the participant: Man vs. Woman) between-subjects design. Participants were randomly assigned to one of two experimental conditions. In the first condition (*n* = 71), they answered a questionnaire with a male uncivil agent, whereas it was a female uncivil agent in the second condition (*n* = 73). Six dependent variables were assessed in each condition: Civility of the behavior (to verify the incivility of the chosen behaviors), Civility of the agent, Moral outrage, Social control, Moral and angry emotions, and Dehumanization of the agent. G*Power 3.1 (Faul et al., 2007) suggests we would need 140 participants in order to detect a medium effect size (*f* = 0.25) with 90% power (α = 0.05).

#### Materials

We created two online surveys with four uncivil situation vignettes each (carried by a man vs. a woman) and six questions (civility of the behavior, civility of the agent, moral outrage, social control, moral and angry emotions, and dehumanization of the agent) for each situation.

##### Uncivil situation vignettes

We selected four behaviors from the same database used in Study 1: damaging the street furniture, not crossing the street through the crosswalk, not picking up the dog’s droppings, and throwing the cigarette butt on the ground. Data were also extracted from the same pre-test (*N* = 28) carried out in Study 1 to determine to what extent behaviors were negative (1) or positive (5) and to what extent they were considered uncivil (1) or civil (5). The results of the one-sample *t*-test showed that the four behaviors were significantly different from the midpoint of the negativity scale (*M* = 1.20; SD = 0.61; *t* (29) = 16.16; *p* < 0.001 for the behavior “damaging the street furniture”; *M* = 1.63; SD = 0.72; *t* (29) = 10.42; *p* < 0.001 for the behavior “not crossing the street through the crosswalk”; *M* = 1.23; SD = 0.43; *t* (29) = 22.49; *p* < 0.001 for the behavior “not picking up the dog’s droppings,” and, finally, *M* = 1.50; SD = 0.93; *t* (29) = 8.76; *p* < 0.001 for “throwing the cigarette butt on the ground”). In addition, they were also significantly different at the midpoint of the civility scale (*M* = 1.30; SD = 0.88; *t* (29) = 10.62; *p* < 0.001 for the behavior “damaging the street furniture”; *M* = 1.87; SD = 1.11; *t* (29) = 5.61; *p* < 0.001 for the behavior “not crossing the street through the crosswalk”; *M* = 1.37; SD = 0.43; *t* (29) = 18.25; *p* < 0.001 for the behavior “Not picking up the dog’s droppings,” and, finally, *M* = 1.67; SD = 0.38; *t* (29) = 26.49; *p* < 0.001 for “throwing the cigarette butt on the ground”).

Subsequently, we hired a professional illustrator to transform the written behaviors into vignettes (see Supplementary Appendix B). The uncivil vignettes were revised by a sample of 16 people, similar to the definitive sample, in order to indicate to what extent it was easy (0) or difficult (10) to understand each of the illustrations and write what they saw in the situation. In this case, the *t*-test showed that the illustrations adequately represented the selected behaviors (*M* = 2.84; SD = 2.19; *t* (18) = 4.29; *p* < 0.001 for the behavior “damaging the street furniture”; *M* = 1.11; SD = 1.82; *t* (18) = 9.31; *p* < 0.001 for the behavior “not crossing the street through the crosswalk”; *M* = 1.25; SD = 1.57; *t* (15) = 9.55; *p* < 0.001 for the behavior “Not picking up the dog’s droppings,” and, finally, *M* = 2.44; SD = 2.87; *t* (15) = 3.56; *p* = 0.003 for “throwing the cigarette butt on the ground”). These illustrations were presented in such a way that the same behavior was done by a female agent in some vignettes and by a male agent in others. In addition, to avoid any doubt, each illustration was accompanied by a legend that identified what the agent was doing (e.g., “You are walking down the street and when you turn the corner you see that there is a man/woman who does not pick up his/her dog’s droppings”).

After seeing each of these behaviors, we asked the participants to pay attention and look at each situation carefully. For each situation, we asked them to rate the civility of the behavior portrayed in the situation, the civility of the agent, the extent to which they will feel certain emotions when facing that situation, how they will react, and what emotions they expect the uncivil agent to feel. Finally, they rated the agent on the Human-Animal scale.

##### Civility of the behavior

After they read each situation, a question was included to verify the civility of the behavior. Specifically, they were asked to rate the civility of the behavior on a seven-point scale with endpoints labeled 1 = *slightly uncivil* and 7 = *very uncivil*. The internal consistency of the four items was acceptable (α = 0.69). We expect men and women participants to rate the uncivil behaviors presented as uncivil.

##### Civility of the agent

The participants also rated the civility of the agent on a seven-point scale with endpoints labeled 1 = *slightly uncivil* and 7 = *very uncivil*. The internal consistency of this score in the four behaviors was equal to α = 0.73. We expect women participants to rate the uncivil agent as more uncivil than men participants (H2a), and for female transgressors to be rated as more uncivil than male transgressors (H2b).

##### Moral outrage

We used the moral emotions presented by Moisuc et al. (2018) to measure the moral outrage that it would entail for the participants to observe a person engaging in such uncivil behavior. Specifically, we asked the participants to rate how intensely they would feel fear, disdain, frustration, anger, sadness, disgust, and shame on a seven-point scale with endpoints labeled 1 = *not intensely at all* and 7 = *very intensely*. The internal consistency of the responses to these items in the four situations was equal to α = 0.93. We expect women participants to experience more moral outrage than men participants (H2c), and for female transgressors to provoke more moral outrage (H2b).

##### Social control

After responding to questions about moral outrage, participants were asked to indicate how they would react on a six-point scale adapted from Chekroun and Brauer (2002); Moisuc et al. (2018). Each point on the scale included one of the following reactions: (1) no reaction; (2) a disapproving look; (3) a loud audible sigh that could be heard by the person; (4) a polite comment to the person, pointing out that the behavior is wrong; (5) an aggressive comment to the person, pointing out that the behavior is wrong; and (6) an insult directed at that person. The internal consistency of the responses to these items in the four situations was equal to α = 0.68. We expect women participants to enact more social control than men participants (H2d), and for female transgressors to elicit more social control (H2b).

##### Moral emotions and angry emotions

This measure recorded the emotional responses that participants estimated that offenders might have if they told them their behavior was wrong. Specifically, following Haidt (2003); Nugier et al. (2007), four moral emotions (guilt, shame, remorse, and regret) and four angry emotions (anger, irritation, upset, and disgust) were included. Thus, the participants had to indicate on a scale from 1 (*not at all*) to 5 (*a lot*) “to what extent do you think that man (woman) could feel these emotions if you told him (her) that the behavior is wrong.” The internal consistency of the four moral emotions was equal to α = 0.92 in the four vignettes and α = 0.90 in the angry emotions. We expect women participants to expect more moral and angry emotions from the agent when called out than men participants (H2d), and for female transgressors to be given more emotional responses (H2b).

##### Dehumanization of the agent

Participants responded to a 0–100 horizontal slide question, where 0 = *human* and 100 = *animal*, where they were asked to place the image they formed about the agent. We expect women participants to dehumanize the agent more than men participants (H2a), and for female transgressors to be dehumanized more (H2d).

#### Procedure and data analysis

We collected data using a self-administered online questionnaire through the Qualtrics platform. Then, we generated an electronic reference for each survey (one survey showed vignettes with a male agent and the other with a female agent) and distributed it randomly to students through the virtual campus of the university. After giving informed consent, the participants viewed the four uncivil situations of each survey in random order and responded to the questions for each situation.

SPSS program 25 version was used for the analyses. A significance level of.05 was set. Descriptive statistics were calculated, and a between-subjects ANOVA of 2 (Agent of the behavior: Male vs. Female) × 2 (Gender of the participant: Man vs. Woman) was conducted with each dependent variable.

### Results and discussion

#### Civility of the behavior

To determine if the participants had perceived the uncivil behaviors presented equally, we conducted a between-subjects ANOVA of 2 (Agent of the behavior: Male vs. Female) × 2 (Gender of the participant: Man vs. Woman) with the civility of behavior as the dependent variable. The results showed that there were no differences due to the agent (*F*(1,140) = 0.250; *p* = 0.618; η*p*^2^ = 0.002; *M* = 5.21; SD = 1.11 when the agent is female and *M* = 5.14; SD = 1.13 when the agent is male) or due to the participant (*F*(1,140) = 3.51; *p* = 0.063; η*p*^2^ = 0.025; *M* = 5.34; SD = 1.16 when the participant is a woman and *M* = 5.00; SD = 1.04 when the participant is a man). Furthermore, the interaction between both variables was not significant (*F*(1,140) = 0.049; *p* = 0.824; η*p*^2^ = 0.00). Taken together, these results show that, regardless of gender, all of the participants rated the behaviors as equally uncivil which confirms the use of the chosen behaviors.

#### Civility of the agent

Even though there were no differences regarding the incivility of the behaviors, significant differences in the participants’ gender were found when asked about the incivility of the agent. Specifically, the between-subjects ANOVA of 2 (Agent of the behavior: Male vs. Female) × 2 (Gender of the participant: Man vs. Woman) conducted with the civility of the agent as the dependent variable showed a main effect of the gender of the participant (*F*(1,140) = 5.70; *p* = 0.018; η*p*^2^ = 0.039). Female participants rated the agent (both female and male) as more uncivil (*M* = 5.34; SD = 1.18) than male participants (*M* = 4.90; SD = 1.07). Neither the sex of the agent (*F*(1,140) = 1.82; *p* = 0.180; η*p*^2^ = 0.013) nor the interaction (*F*(1,140) = 0.85; *p* = 0.357; η*p*^2^ = 0.006) was significant.

In summary, when facing uncivil behavior, both men and women give the same degree of incivility to the behavior. However, when asked about the agent that carried the uncivil behavior, women rated the transgressor as more uncivil than men which are in line with Hypothesis 2a.

#### Moral outrage

The between-subjects ANOVA of 2 (Agent of the behavior: Male vs. Female) × 2 (Gender of the participant: Man vs. Woman), which we executed to determine the moral outrage that participants could feel if they observed the uncivil behaviors presented in the vignettes, resulted in the main effect of the gender of the participant (*F*(1,140) = 13.26; *p* < 0.001; η*p*^2^ = 0.086). Female participants reported more moral outrage (*M* = 3.93; SD = 1.14) than male participants (*M* = 3.27; SD = 1.06). Whereas, the gender of the agent was not significant (*F*(1,140) = 1.06; *p* = 0.305; η*p*^2^ = 0.007). Interestingly, there was an interaction between the gender of the participants and the agent of the behavior (*F*(1,140) = 3.99; *p* = 0.048; η*p*^2^ = 0.028). The pairwise comparisons showed that when a female is the uncivil agent, female participants (*M* = 4.22; SD = 0.18) felt more moral outrage than male participants (*M* = 3.19; SD = 0.18; *t* (140) = 4.02; *p* < 0.001; Cohen’s *d* = 5.72). On the other hand, when a male is the uncivil agent, there are no differences in the moral outrage felt by female (*M* = 3.67; SD = 0.17) and male participants (*M* = 3.37; SD = 0.19; *t* (140) = 1.15; *p* = 0.252; Cohen’s *d* = 1.47). This means, there is a difference between men and women when feeling moral outrage after facing uncivil agents, being that women report more moral outrage than men, which is in line with Hypothesis 2c. However, the interaction found plays a big role here since there are no disparities between men and women when they are facing an uncivil male, but when it is an uncivil female, female participants reported more moral outrage than male participants, which is partially in line with Hypothesis 2b.

#### Social control

The mean score on the social control scale was subjected to a between-subjects ANOVA of 2 (Agent of the behavior: Male vs. Female) × 2 (Gender of the participant: Man vs. Woman). The results showed a main effect of the gender of the participant (*F*(1,140) = 5.90; *p* = 0.016; η*p*^2^ = 0.040). Female participants (*M* = 2.66; SD = 0.88) tend to respond with behaviors that involve greater social control than male participants (*M* = 2.30; SD = 0.88). On the other hand, no significant differences where observed regarding the gender of the transgressor (*F*(1,140) = 0.57; *p* = 0.452; η*p*^2^ = 0.004) or the interaction (*F*(1,140) = 0.91; *p* = 0.341; η*p*^2^ = 0.006).

#### Moral emotions and angry emotions

The ANOVA performed with the responses to moral emotions showed a main effect of the gender of the participant (*F*(1,140) = 8.22; *p* = 0.005; η^2^ = 0.055). Female participants (*M* = 2.87; SD = 0.86) expect more moral emotions from uncivil transgressors when called out, than male participants (*M* = 2.48; SD = 0.78). No significant differences were found regarding the gender of the transgressor (*F*(1,140) = 1.93; *p* = 0.167; η*p*^2^ = 0.014) or the interaction (*F*(1,140) = 0.62; *p* = 0.431; η*p*^2^ = 0.004). The analysis of the angry emotions produced the same result. That is, the gender of the participant was significant (*F*(1,140) = 8.03; *p* = 0.005; η^2^ = 0.054). Women (*M* = 3.24; SD = 0.83) tended to expect more angry emotions from uncivil transgressors when called out, than men (*M* = 2.88; SD = 0.74). No significant differences were found regarding the gender of the transgressor (*F*(1,140) = 2.04; *p* = 0.155; η*p*^2^ = 0.014) or the interaction (*F*(1,140) = 1.46; *p* = 0.229; η*p*^2^ = 0.010).

Women enact more socially controlling behaviors than men. This is, when facing uncivil agents, women react more than men, expecting a response from them which is in line with the fact that they expect more moral and angry emotions from the transgressors when they are criticized, which is in line with Hypothesis 2d.

#### Dehumanization of the agent

The between-subjects ANOVA of 2 (Agent of the behavior: Male vs. Female) × 2 (Gender of the participant: Man vs. Woman), performed to determine the degree of dehumanization of the agent of the uncivil behaviors gave rise to the main effect of the gender of the participant (*F* (1,138) = 4.70; *p* = 0.032; η*p*^2^ = 0.033). Female participants (*M* = 48.87; SD = 19.75) tended to associate uncivil agents with animals more than male participants (*M* = 40.77; SD = 24.62), which is in line with Hypothesis 2a. No significant differences were found regarding the gender of the transgressor (*F*(1,140) = 0.167; *p* = 0.684; η*p*^2^ = 0.001), or the interaction (*F*(1,140) = 2.17; *p* = 0.143; η*p*^2^ = 0.016).

To sum up, these results are in line with our hypothesis. When facing uncivil transgressors, women evaluate the transgressor as more uncivil and dehumanize more than men (Hypothesis 2a). And, women report more moral outrage than men (Hypothesis 2c). Moreover, women enact more social control and expect more emotions from the transgressor when they are called out than men (Hypothesis 2d). Finally, a difference based on the gender of the transgressor was observed only with moral outrage (partially Hypothesis 2b) where women are more outraged when the transgressor is another woman. It seems that not only are these supposedly equally applied social norms associated differently with male and female characteristics (Study 1), but the way we react to those who transgress them is also affected by gender, leading to inequality.

## General discussion

The aim of the present study is to explore the relation between norms regarding civility and gender stereotypes. We carried out two studies to explore how civil and uncivil behaviors are associated with stereotypically masculine and feminine traits, as well as how a lack of civility may trigger dehumanization, resulting in consequences that may differ for the transgressors based on their gender.

In Study 1, we asked about an explicitly gender-neutral extraterrestrial society, and the results showed that when asked about a society, which is not even from planet Earth, there were no differences between the characteristics associated with agents that carry out civil and neutral behaviors. However, in the case of uncivil behaviors, it is different. Those who carry out these types of behaviors were associated more with stereotypically masculine characteristics than stereotypically feminine ones, as we stated in Hypotheses 1a. This result is consistent with previous research that found gender differences in counternormative behaviors. Specifically, there seems to be a pattern that verifies that minor transgressions such as committing traffic violations are more associated with men than women. Baxter et al. (1990); Corbett and Simon (1992); Verkuyten et al. (1994), and also, men are more likely to steal than women at the workplace (Ruggiero et al., 1982; Miethe and McCorkle, 1998). However, surprisingly, the results did not allow us to confirm hypothesis 1b. That is, feminine stereotypical traits did not carry a higher association with civil behavior because the differences appeared just in the uncivil behavior. One explanation for this result could be that in a normative and regular context (as in the civil and neutral condition) the stereotypical differences between gender could not be salient enough (Eagly, 2009). Results also showed that participants dehumanize the agent of uncivil behaviors to a greater extent, considering them closer to animals than humans (hypothesis 1c). Just subtracting one of the uniquely human traits, in this case the civism, had enough impact to consider those who commit the transgression as less human. Also, the results yielded that the stereotypically masculine and feminine characteristics mediate the dehumanization of the uncivil agent (hypothesis 1d). Specifically, those who behave uncivilly were dehumanized, but this is only predicted through the lack of stereotypically feminine characteristics. So, the more uncivilly you behave, the more stereotypically masculine you are, but dehumanization will not happen because of an excess of stereotypically masculine traits but because of a lack of stereotypically feminine characteristics (Hypothesis 1d). This raises a concern about gendered expectations in our society.

It seems that civility norms are not equally associated with male and female characteristics. It is understandable since men are usually more related to these norm violations than women, even being seen as positive for the concept of masculinity (Coates, 1999). It is concerning that the lack of femininity is what predicts dehumanization in incivility since stereotypically masculine characteristics are high in these behaviors. This means that incivility leads to dehumanization, but it is not the stereotypically masculine traits that predict it but the lack of femininity in it. With this in mind, it is important to think about how a lack of stereotypically feminine traits can affect a woman but not necessarily a man, given the congruity with the male stereotype and incongruity in the female stereotype, so we conducted Study 2 to test how our gender affects the way we react to male and female uncivil transgressors.

In the second study, we observed that when facing a male and female uncivil transgressor, the responses given by male and female participants were different. Though both men and women gave the same grade of incivility to the behavior, when asked about the uncivil agent that carried out the behavior, female participants saw the transgressor as more uncivil than men (hypothesis 2a). Thus, women dehumanize them to a greater extent than men (hypothesis 2a). Men are aware of their engagement with transgressions, and they also associate stereotypically masculine characteristics with incivility, so they will not be as severe with uncivil transgressors as women. Also, when facing uncivil transgressors, women experienced more moral outrage than men (hypothesis 2c). Moreover, our findings showed that women enact more social control and expect more emotions from the transgressor when they are called out than men (Hypothesis 2d). Finally, a difference based on the gender of the transgressor was observed only with moral outrage where women were more outraged when the transgressor is another woman (hypothesis 2b). These results are in line with the data obtained in Study 1 and with previous research that observed how peers usually act as controllers of gender stereotypes to prevent deviant gender-based behaviors (Lee and Troop-Gordon, 2011). Thus, since women engage in less incivility (Alexander-Snow, 2004) and are expected to behave politely, they are more severe when these social norms are transgressed. Also, these results are in line with the findings of Gabriel et al. (2018); Sheppard and Aquino (2017), where, in workplace context, women who do not reproduce gender stereotypes and do not meet the social expectations of their ingroup members (i.e., other women), face a penalty.

Generally, women are more aware of discrimination than men (Basford et al., 2014), and it has been seen in the case of civility that when incivilities are carried out in organizations, the uncivil ones are often men, and women are frequently the ones who suffer from incivility (Cooper et al., 2013; Porath and Pearson, 2013; Loi et al., 2015). Moreover, there is a strong stereotype about women, considering them as those who have concern for the welfare of others, display kind behaviors, being cooperative, nurturing and gentle (Sheppard and Aquino, 2017). However, there are consistent social consequences when women violate those stereotypes (Heilman et al., 2004). So, non-communal behaviors in women can lead to the black sheep effect, and the ingroup (women) could perceive that behavior as a collective threat and react negatively (Gabriel et al., 2018). This fact explains why, in our results, women were harsher on the female uncivil agent given the incongruity of the behavior with the feminine role (H2b), thus reporting more moral outrage in the case of the female uncivil target. In fact, according to Gabriel et al. (2018), low levels of communion will be more likely to experience female-instigated incivility. The moral outrage could be related to these negative reactions from the ingroup (women participants), given that aversion emotions are associated with intergroup threats. Finally, research pointed out that women seem to be proud of their communal stereotypical traits (Glick and Fiske, 1996, 2001) and this fact allows them to recognize and differentiate themselves positively from men (Brewer, 1991). Thus, their ratings in social control, negative emotions, dehumanization, and incivility, could be the consequence of the lack of communion of others.

These gender differences in civility and incivility show a worrying consequence of the socialization process. Norms regarding civility are established to help individuals, showing them how to interact correctly with other members of their community. These norms are learned through the socialization process. However, this process also teaches them how to behave under gender norms, and these norms are neither perceived nor applied by us equally to other men and women, resulting in this twist on social norms. The inequality that happens from gender stereotypes has serious consequences, especially for women, because it often leads to power relations that do not bring them benefits (Lazar, 2005; Connell, 2014). They have to respect the traditional masculine role, which often puts them in a disadvantageous position. Also, they need to strongly follow feminine stereotypes that are more focused on support, being tactful, and not stepping out of line, since women who do not conform to stereotypically appropriate behaviors will be seen as carrying deviant behaviors, which, albeit minor, can lead to harsh penalties and sanctions, even prison time (Carlen, 1998). It is worrisome that this difference is not present only in negative behaviors. Even when a woman carries a polite behavior, but such behavior is not consistent with a feminine stereotype, she will be subjected to criticism, whereas the same consequence does not happen when it is a man (Sung, 2012).

Gender inequality not only affects women negatively, but these roles can also encourage men to behave in a way that may seem positive but can be detrimental to them. Coates (1999) states that when “bad” behaviors are implicated, such as talking publicly about pornography, it is seen as negative if it is done by women but positive if it is done by men. This positive encouragement of certain behaviors is not only present in adults. Young boys who are exposed to and influenced by their peers seem to be more resistant to schooling than girls (Geven et al., 2017), and male adolescents who follow and conform to masculine gender identity are more prone to school misconduct than females (Heyder et al., 2021). If males are expected to behave in certain ways, but those ways are not in line with societal norms, it may lead to them giving less importance to those transgressions through a process of moral disengagement or moral rationalization to maintain their personal image, which may be what happened in the case of incivility. The normalization of male incivility not only affects their own perception but also undermines the wellbeing of the members of their society. In this way, dominant norms of masculinity can result in harm for both men and women (Courtenay, 2000; Connell and Messerschmidt, 2005; Evans et al., 2011).

It is important to note that the differences between men and women were found in actual real situations and that asymmetry is also present in our minds. Moreno-Bella et al. (2019) found that, in highly economically unequal societies, we associate upper-class people with more masculine traits than feminine ones. So, these differences are not only experienced but also projected by us. Thankfully, these gender differences were not left unseen. The cultural impact of the Women’s Movement and the wave of feminism have led to a reevaluation of the ideas of femininity and how women are defined in our culture–as well as men’s expectations of manhood and how they view themselves and others as men–all working toward a less gender-biased and more civil society (Miller, 2002).

The results presented here are relevant in various ways. First, they support and contribute to the evidence that gender norms, even if they are related to positive attributes such as being more competent or warm, can end in differences that support stereotypes, leading individuals to evaluate others in a gendered manner, even when they are clearly stated as gender-neutral. Entities that only share metaphorical similarities with men and women are perceived to be masculine or feminine even though there are no female and male sex categories (Bem, 1981; Starr and Zurbriggen, 2017). However, our results raise concerns in our society because not only it is harsh on men and women but clearly shows that even those who clearly define themselves as gender-neutral, are still judged by gender norms and stereotypes depending on their behaviors. In this way, behaving uncivilly is stereotypically masculine, but the consequences of uncivil transgressions differ between men and women unequally. Second, it shows that uncivil transgressions, which are pretty common, lead to dehumanization, a negative consequence. Not only that, this dehumanization is very related to a lack of stereotypically feminine characteristics in this case. Third, it is remarkable the punctuation of moral outrage reported for women participants facing female uncivil behavior. This fact could be related to the women’s stereotypes violation. That is, society expects women to be communal and when an ingroup member shows counternormative behaviors, the ingroup can penalize this behavior. Fourth, negative stereotypes about men can lead to positive outcomes for men. The fact that men are being related to minor moral and legal violations does not mean they will be judged more harshly because of it. Alternatively, since women are not that related to incivility, when they transgress, they will receive more outrage from fellow women. In the association between civility and gender, the valence of a stereotype does not equal the valence of the consequence. this study contributes to and extends the literature on gender and incivility to general contexts. Most of the research on this topic is focused on specific contexts such as organizational contexts with similar results (i.e., women rated harder to other women than men; Gabriel et al., 2018) but this pattern seems to be extending to everyday life and is asymmetrically associated with stereotypical gender traits.

This research also had some limitations that should be considered when interpreting the results. The first limitation is the sample type of both studies, which were composed of undergraduate students, so it would be interesting to carry out these studies with the general population. Second, both studies use hypothetical scenarios and vignettes that may differ from real-life interactions, so although the two present experiments had high internal validity, they may lack ecological validity. Finally, even though the studies were conducted with daily civil and uncivil behaviors, we used a small sample, so it is necessary to try more civil and uncivil behaviors.

In conclusion, even when civility norms are supposed to be applied equally to individuals in our society, today’s civil and uncivil behaviors are unequally associated with masculine and feminine stereotypes, leading to differences in the perception of incivility between men and women, and divergence when facing uncivil transgressors based on gender. Incivility is more associated with stereotypically masculine characteristics, but men will not receive a harsher punishment. It is women who react more strongly in the face of uncivil transgressors, especially if the said transgressor is another woman. Everyone is expected to act out their gender role and follow the civility norms of their society, but not everyone who disregards those roles and norms will be judged equally. Future research should also consider other variables (e.g., stereotypes that each individual carries, age, or culture) that may be influencing the perception of incivilities, as well as which processes may be mediating the evaluation of uncivil transgressors.

## Data availability statement

Data used for this study are available upon request to the corresponding author.

## Ethics statement

The studies involving human participants were reviewed and approved by Ethics Committee on Research and Animal Welfare of the University (CEIBA). The patients/participants provided their written informed consent to participate in this study.

## Author contributions

XC-X contributed to data curation, formal analysis, investigation, methodology, software, visualization, and writing – original draft, review and editing. VB contributed to project administration, conceptualization, funding acquisition, supervision, and writing – review and editing. AC contributed to supervision, and writing – review and editing. AR-P contributed to conceptualization, funding acquisition, investigation, project administration, resources, supervision, and writing – original draft, review and editing. All authors contributed to the article and approved the submitted version.

## Footnotes

1 Rodríguez-Gómez, L., Delgado, N., Betancor, V., Rodríguez-Torres, R., and Rodríguez-Pérez, A. (Under review). Social perceptions of civility and their link with dehumanization theory.
