# Supplementary material for: Gender inequality in incivility: Everyone should be polite, but it is fine for some of us to be impolite
Source: Front Psychol. 2022 Sep 26;13:966045. doi: 10.3389/fpsyg.2022.966045 (PMC9549928; doi:10.3389/fpsyg.2022.966045)
Supplement: Supplementary file 1 [file Presentation_1.pdf]

Supplementary Material

Appendix A

Background story: "Imagine an extraterrestrial society - the Ortandesíes - in which the inhabitants can be considered neither male nor female. Their reproductive system is neutral, so anyone can breed with any other individual. This society has existed for centuries. Individuals have scaly skin and two rows of teeth, and other attributes vary considerably among inhabitants. In addition, they have a language without vowels with only two sounds, F and M, so they communicate everything through different combinations of those two letters. The members of this society live in large cities like those that exist on earth and enjoy their free time walking in parks and many entertainment places, so they have various opportunities to have fun, both individually, with their pets, relatives, or other Ortandesíes. In addition, in this society there are norms of coexistence and respect for others to harmonize life in the community, but, as is the case in most, there are Ortandesíes who behave civilly and Ortandesíes who behave uncivilly."

Original version (Spanish): "Imagínese una sociedad extraterrestre – los Ortandesíes - en la que los habitantes no pueden ser considerados ni mujeres ni hombres. Su sistema reproductivo es neutral, por lo que cualquiera puede reproducirse con cualquier otro individuo. Esta sociedad existe desde hace siglos. Los individuos tienen piel escamosa y dos filas de dientes, y el resto de atributos varían considerablemente entre los habitantes. Además, tienen un lenguaje sin vocales con solo dos sonidos la F y la M de modo que todo lo comunican a través de distintas combinaciones de esas dos letras. Los miembros de esta sociedad viven en grandes ciudades como las que existen en la tierra y disfrutan de su tiempo libre paseando en los parques y en muchos lugares de ocio, de modo que tienen varias oportunidades para divertirse, tanto individualmente, como con sus mascotas, familiares

u otros Ortandesíes. Además, en esta sociedad existen normas de convivencia y de respeto a los otros para poder armonizar la vida en comunidad, pero, como ocurre en la mayoría, hay Ortandesíes que se comportan de forma cívica y Ortandesíes que se comportan de forma incívica."

Spanish adaptation of the BSRI (Bem, 1974; adapted to Spanish by Páez and Fernández, 2004):

Masculine traits	*Feminine traits*
Atlético/a, deportivo/a	Cariñoso/a
Personalidad fuerte	Sensible a las necesidades de los demás
Desea arriesgarse, amante del peligro	Comprensivo/a
Dominante	Compasivo/a
Agresivo/a, combativo/a	Cálido/a, afectuoso/a
Actúa como líder	Tierno/a, delicado/a, suave
Individualista	Amante de los niños
Duro/a	Alguien que llora fácilmente
Egoísta	Sumiso/a

Appendix B

Men vignettes used in the first experimental condition of Study 2

Damaging the street furniture	Not crossing the street through the crosswalk	Not picking up the dog's droppings	Throwing the cigarette butt on the ground
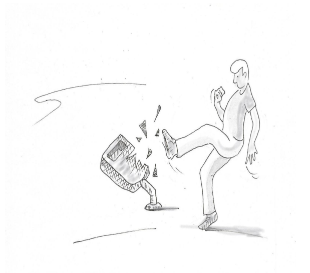	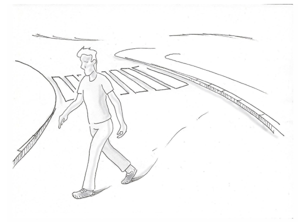	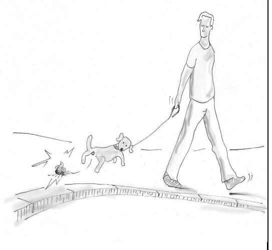	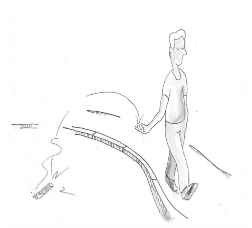

Women vignettes used in the second experimental condition of Study 2

Damaging the street furniture	Not crossing the street through the crosswalk	Not picking up the dog's droppings	Throwing the cigarette butt on the ground
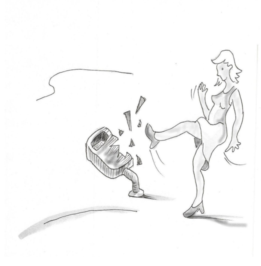	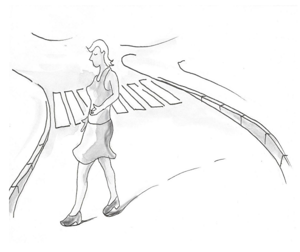	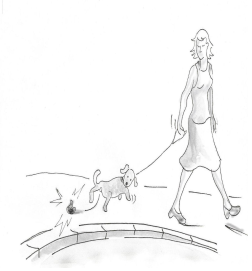	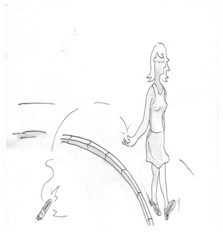
